# A New Dietetic and Injection Method of Treating Typhoid Fever

**Published:** 1908-11

**Authors:** 


### ABSTRACT.

### A New Dietetic and Injection Method of Treating Typhoid Fever

**With a Report of One Hundred and Thirty-Eight Consecutive Cases Successfully Treated in the Last Ten Years**

F. J. W. Maguire (*Journal of Michigan State Medical Society*, July, 1908), under the above title states his conclusions upon experience gained in the United States Marine Hospital service and in private practice. In part, he says:

I noticed when treating children with summer diarrhea that shortly after giving them nitrogenous food in the form of milk or beef tea their temperature would always rise. I found that by giving these children a carbohydrate diet in the form of barley or rice water, I rarely had a rise in temperature. With this observation in mind and remembering the results found in my autopsies following typhoid, I came to the conclusion that milk as a diet in typhoid fever should be eliminated. To further strengthen this theory, I determined to carefully watch the results following the use of carbohydrate diet in the form of rice or barley water and the like. In eighteen cases I found the temperature rise following the milk diet, while there was no perceptible increase in temperature after taking rice or barley water.

I need scarcely add that as a food in typhoid fever I have never since used milk. It is my practice, when I first see a typhoid fever case, to give plenty of sterile water by mouth for five to ten days or until the patient seems to require nourishment, then I use the peptonoids well diluted with sterile water, and the various flavored ices and gelatines. I condemn cow's milk, as it is a culture medium and the cause of a great deal of local irritation.

With reference to treatment the author states:

Having eliminated the milk diet with its terrible irritating effects in the already inflamed Peyer's patches, half the battle is won. This brings us to a consideration of the therapeutic aspect of this subject. In taking up the use of carbolic acid as the therapeutic agent in typhoid fever, I at first thought that I had discovered means whereby I could abort the disease. I commenced by giving half-dram doses of carbolic acid in a pint of sterile water as an enema. This I found very severe. The temperature would drop from 104 to subnormal and the patient showed signs of carbolic acid poisoning. The temperature would run from normal to 100 for a few hours; then resume its course. The kidneys were carefully watched in all these cases, as they are the filters by which the toxins are eliminated. In my next series of experiments I began with one drop of carbolic acid in a pint of sterile water given as an enema; if the temperature was not reduced I gave another enema in three hours with two drops, and so on increasing until I gave as high as ten drops or the tolerance of my patient allowed. My next series of experiments was with the drop method of injection. I mixed three to five drops of carbolic acid in a pint of sterile water, placed the solution in a fountain syringe alongside the bed and about a foot above the patient, and allowed about one hour for the solution to pass into the rectum. This was regulated by a gauge with a water glass attachment which shows how fast the water drops. Through the reverse mucous currents this solution is carried throughout the intestinal tract and through this large area of absorption is carried to every tissue in the body.

In conclusion the author says:

I do not limit the use of carbolic acid injection to typhoid treatment in reducing temperature in pneumonia and gastritis and have carried cases of acute appendicitis to a sub-acute or chronic form, thereby lessening the danger from infection at the time of operation. In these 138 cases reported here today, the ages ranged from three to seventy-eight years. I gave no cold baths, but applied ice bags over abdomen, and one bath a day for cleanliness. Occasionally I gave a little strychnine, quinine and salol as indicated. Since adopting this dietetic and carbolic injection method of treating typhoid fever, I have treated 138 consecutive cases. This covers a period of about ten years. All these cases responded readily to treatment, notwithstanding the fact that many were advanced before treatment was begun. Four cases had had most profuse hemorrhages, all of which subsided when the milk diet was removed. I believe by these experiments, I have made some very valuable therapeutic and dietetic discoveries, and have sufficient confidence in my treatment that I am compiling a work on the subject.

